# Cobalt (II)-Mediated Molecularly Imprinted Polymer as a Monolithic Stationary Phase for Separation of Racemic Citronellal by Liquid Chromatography

**DOI:** 10.1155/2022/7891525

**Published:** 2022-02-28

**Authors:** Suci Amalia, Nilna Assasiatur Rafika, Shova Audinia Hardiyanti, Adi Dwi Ashari, Bhisma Wildan Khabibi, Elvina Dhiaul Iftitah, Warsito Warsito, Aliya Nur Hasanah, Akhmad Sabarudin

**Affiliations:** ^1^Department of Chemistry, Faculty of Science, Brawijaya University, Malang 65145, Indonesia; ^2^Department of Chemistry, Faculty of Science and Technology, Islamic State University Maulana Malik Ibrahim, Malang 65144, Indonesia; ^3^Pharmaceutical Analysis and Medicinal Chemistry Department, Faculty of Pharmacy, Universitas Padjadjaran, Sumedang 45363, Indonesia; ^4^Research Center for Advanced System and Material Technology (ASMAT), Brawijaya University, Malang 65145, Indonesia

## Abstract

A metal-mediated molecularly imprinted polymer (MMIP) monolithic column was prepared as the stationary phase for high-performance liquid chromatography (HPLC) and applied to the enantiomeric separation of *rac*-citronellal. MMIP column was prepared through in situ copolymerizations with the ionic liquid 1-butyl-3-methylimidazolium tetrafluoroborate/[BMIM][BF_4_] as the primary pore-forming agent and cobalt(II) acetate as the metal pivot. Interactions between polymer components in the synthesized monolith were assessed by FTIR to identify the functional groups. The monolith morphology was characterized with SEM, and the template removal was detected by UV Spectrophotometry at 253 nm. In this study, (R)-(+)-citronellal was used as a template, whereas 4-vinylpyridine (4-VP) was employed as the functional monomer with two monomer crosslinkers, trimethylolpropane trimethacrylate (TRIM), and ethylene glycol dimethacrylate (EDMA). The ternary mixture of porogenic solvent consisted of [BMIM][BF_4_], dimethylformamide (DMF), and dimethyl sulfoxide (DMSO) with the applied ratio of 1 : 1:1 (v/v) and 10 : 1:5 (v/v) for the preparation of MMIP using TRIM and EDMA crosslinkers, respectively. Co(II) ion was added to the porogenic solvent before being mixed with the functional monomer and the crosslinker mixtures. Separating the *rac*-citronellal was achieved on the synthesized MMIP, showing better selectivity than the monolithic metal-mediated nonimprinted polymer (MNIP), nonimprinted polymer (NIP), and molecularly imprinted polymer (MIP).

## 1. Introduction

Monoliths have an excellent ability to separate isomers, especially as chiral stationary phase (CSP) in high-performance liquid chromatography (HPLC). They have porous structures with sizes between micro and macro; thus, they may provide good pressure reduction and increase the mass transfer kinetics facilitating higher flow rates [1, 2]. Additionally, monolith materials could also reduce the length of the diffusion path while providing flow resistance [3–5].

Molecularly imprinted polymer (MIP), as one of the techniques for the synthesis of stationary phases for chromatography, is increasingly in demand for separating enantiomer compounds [6–9]. The stationary phase is prepared by a hollow polymer formation after template removal from the polymer structure. Accordingly, such a technique offers high selectivity due to the hollow parts that will recognize molecules in the shape and size of the template [10, 11]. MIP is further developed by adding metal ions into the solution mixture, acting as a bridge between the functional monomer and the template via coordinate bonding. Using metal pivot will keep the monomer in the template vicinity by preventing the free motion of functional groups involved in the reaction, by decreasing nonspecific binding sites, and by increasing selectivity towards analytes [12, 13]. This later technique is called a metal-mediated imprinted polymer (MMIP).

Metal ions including Co (II), Cu (II), Zn (II), and Ni (II) have been recognized for their use in MMIP preparation applied to the separation of racemic mandelic acids [10]. Sun et al. [14] demonstrated that Zn (II), implemented as a metal center in the extraction of chicoric acid from the roots of *Cichorium intybus* L., could be used to overcome a weak hydrogen bond between the template and functional monomer. A longer retention time is required to obtain chicoric acid than its analogs. Moreover, the purification of methyl gallate was carried out with MIP, as reported by Zhong et al. [15]. It was found that adding a template and Co(II) for the synthesis of the stationary phase leads to a longer retention time when methyl gallate is eluted. Based on these results, Co(II) is deemed as the crucial factor in the separation process.

In addition, Feng et al. [16] reported that an increase in the imprinting factor (IF) of MIPs is a present manifestation of Co(II) ion. The synthesis of MIP PMMA-S-NAP and S-KET resulted in IF 1.26 and 1.01. In contrast, the IF that resulted from MIP Co(II)-PMMA-S-NAP and S-KET was 2.10 and 1.90, respectively, leading to a successful synergistic effect in printing activity. Wei et al. [17] reported that cetirizine content in ethanol was extracted with a recovery yield of 97.8%. The extraction was conducted with MIP monolith where Co (II) was a chelating agent, resulting in an imprinting factor 23.5 times higher than that without Co (II). Almost all bivalent metals prefer complexes with a coordination number of 6 or an octahedral shape. The formation of octahedral complexes by bivalent metals with 3d^n^ configuration is high-spin (weak-field complex) or low-spin (strong-field complex) for *n* = 4–7. Furthermore, transition metals with valence >7 coordinate to form strong-field complexes only. According to Ligand-Field Stabilization Energy (LFSE) [18], the highest stability was achieved by metal ions with configuration d^7^ as its LFSE = 1.8 Δo-P (Δo = the ligand-field splitting parameter, *P* = pairing energy). The order of octahedral complex stability is as follows: Co (II) > Ni (II) > Cu (II) > Zn (II) according to their electron configurations d^7^, d^8^, d^9^, and d^10^, respectively. It means that the Co(II) can form the most stable strong-field octahedral complex with the N donor atom of the 4-VP functional monomer [18], resulting in better IF for the MMIP preparation.

The use of ionic liquid (IL) as a solvent in polymer synthesis progressively grows due to several excellent chemical properties, such as high conductivity, nonflammability, thermal stability, and low vapor pressure at room temperature [19, 20]. IL also exhibits unique solvation properties to perform discrete polar and nonpolar microenvironments. These properties solve the solubility problems when using conventional organic solvents [21]. Furthermore, IL can also accelerate the polymerization reaction [22].

DMF-DMSO-IL solvent mixture is highly recommended for the synthesis of monolith organic polymer, mostly for a polar template. In the synthesis process, DMF and DMSO dissolve the metal pivot and regulate template retention in MMIP and MIP. The use of DMSO-combined IL as a porogen will provide good solvation quality so that the detailed printing effect can be achieved [23]. The porogenic solvent also serves to strengthen complex structure monolith through polymerization [15].

In this work, MMIP was synthesized using Co(II) ions as the pivot of metal assembly. The solution mixture consisted of (R)-(+)-citronellal template, functional monomer (4-VP), and crosslinker (TRIM) or (EDMA) with different ratios of porogenic solvent. The porogenic solvent was prepared from a mixture of [BMIM] [BF_4_] ionic liquid, DMF, and DMSO. The method used in the MMIP monolith synthesis was metal-mediated molecular self-assembly (MMSA). The combination of these materials was intended to produce the polymeric stationary phase with high sensitivity and selectivity in separating enantiomeric compounds based on materials' characteristics. The MMIP monolith in this work was prepared by in situ copolymerization inside silicosteel® tubing (100 mm length × 1.0 mm i.d). Then, it was applied as chiral stationary phase (CSP) in HPLC to separate and purify rac-citronellal compounds by observing several tested parameters, such as the composition and concentration of materials, column performance, separation process, flow rate, and mobile phase composition. Furthermore, the physical characteristic of the monolithic column was also considered through characterization by several instruments such as a UV-Vis and Fourier Transform Infrared (FTIR) Spectrophotometers and Scanning Electron Microscope (SEM).

Although we apply similar monomers, porogen, and metal pivot, the MMIP polymerization in this study was carried out in situ inside a 1.0 mm i.d. microbore silicosteel column with the template of (R)-(+)-citronellal, which is smaller than those employed by Bai et al., 4.6 mm i.d. with the template of mandelic acid [10], and Bodoki et al., 2.1 mm i.d. with the template of atenolol [12]. The different templates strongly influenced the selectivity of the columns in the MMIP. Furthermore, it was proved that a small diameter monolithic column would have various advantages over a big one, including greater homogeneity and separation efficiency, and it is environmentally friendly due to the decreased sample and reagent use. Additionally, larger diameter monolithic columns are less homogeneous, not only due to differential heating across the tube diameter but also because of the rising gravitational settling effect during the exothermic polymerization process [1, 24].

## 2. Experimental

### 2.1. Chemicals and Materials

Ethylene glycol dimethacrylate 98% (EDMA) and trimethylolpropane trimethacrylate 90% (TRIM) of Tokyo Chemical Industry (TCI), Japan, were applied as crosslinking agents, while the 4-vinyl pyridine (4-VP) 95% from TCI (Japan) was used as the functional monomer. The porogenic solvent consisted of N,N-dimethylformamide 99.8% (DMF), dimethyl sulfoxide ≥99.9% (DMSO), and 1-butyl-3-methylimidazolium tetrafluoroborate ≥98% [BMIM] [BF_4_] ionic liquid, 3-(trimethoxysilyl) propyl methacrylate (MAPS) 98%, pyridine anhydrous 99.8%, sodium acetate anhydrous ≥99%, sodium hydroxide (NaOH) 97%, cobalt acetate tetrahydrate Co(Oac)_2_·4H_2_O 99.99% for analysis, acetonitrile (ACN) for liquid chromatography, acetic acid (glacial) 100%, *α*,*α*′-azobisisobutyronitrile 12 wt% in acetone (AIBN) as the radical initiator, (R)-(+)-citronellal 90%, and (S)-(-)-citronellal 96% which were purchased from Sigma-Aldrich (Singapore). Methanol for GC 99.9% was purchased from J. T. BAKER (USA). Acetone 99.75% was purchased from Mallinckrodt chemical (USA). Water (Aqua Pro Injection) from Ikapharmaindo (Indonesia) and hydrochloric acid (HCl) 37% from PT Smart Lab (Indonesia) were used for reagent preparation. Fused silica silicosteel® tubing (100 mm in length, outer diameter 1/16 inch, and 1.0 mm inner diameter) from Supelco was utilized as a column housing. All materials were high purity and used directly without further purification.

### 2.2. Instrumentation

The chromatographic system applied in this work was an HPLC prominence 20 from Shimadzu equipped with LabSolution software. It consisted of degasser units (DGU-20A), pumps (LC-20AD), oven (CTO-20A), UV-Vis detector (SPD-20A), a semi-micro flow cell (2.5 *μ*L), a Rheodyne 7125 injector with 2 *μ*L loop sample, and a controller (CBM-20A). FTIR-8400S of Shimadzu was used for the identification of functional groups in monoliths. Scanning Electron Microscope (SEM), Hitachi FE-SEM 1000, was used for the morphological imaging, and UV-Vis Spectrophotometer-1601 Shimadzu was employed for the template and Co (II) removal identification.

### 2.3. Preparation of Fused Silica Silicosteel^®^ Tubing as a Monolithic Column Housing

Silicosteel® tubing was pretreated through silanization prior to use [1, 25, 26]. The first step was to rinse the inside silicosteel® tubing with water three times. Afterward, the column was filled with 0.2 M NaOH and left to stand for 30 min, followed by rinsing with water, and subsequently filled with 0.2 M HCl and left to stand 30 min. These procedures were repeated twice. After being treated with NaOH and HCl, the tubing was washed with water three times, followed by acetone. All filling and washing processes were carried out with a syringe. The activated silicosteel® tubing was then filled with 30% MAPS in acetone and pyridine at a ratio of 30 : 65 : 5 (MAPS: acetone: pyridine, v/v). Both ends of the column were closed and left at room temperature for 12 h. This procedure was repeated twice. In the final step, the silicosteel® tubing was rinsed with acetone, cut 100 mm in length and 1 mm i.d, and ready to be filled with the monomer mixture for the monolith polymer synthesis.

### 2.4. Synthesis of Metal-Mediated Molecularly Imprinted Polymer (MMIP) Monolith inside Silicosteel^®^ Tubing

**Figure 1:**
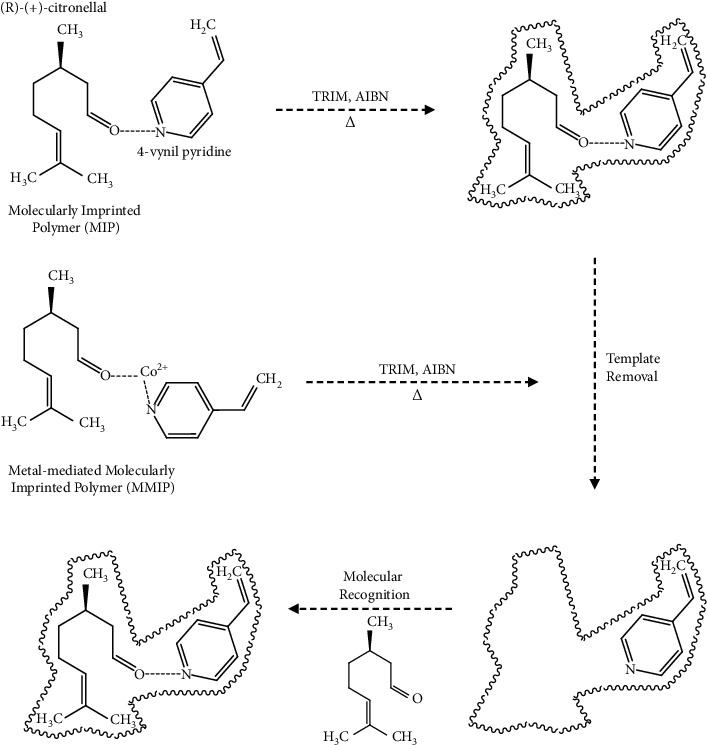
Schematic representation of MIP preparation and MMIP using Co(II) ion as a metal pivot for (R)-(+)-citronellal recognition.

The schematic representation of MMIP preparation with template and metal pivot Co (II) ion in the monolith framework can be seen in Figure 1. In this work, two kinds of MMIPs with different crosslinkers (TRIM and EDMA) were prepared, as shown in Table 1. Ionic liquid [BMIM] [BF_4_] was added with porogen ratio of [BMIM] [BF_4_] : DMF : DMSO 1 : 1:1 and 10 : 1:5 (v/v) for TRIM and EDMA crosslinker, respectively. Afterward, 4-VP functional monomers, (R)-(+)-citronellal template, TRIM/EDMA crosslinker monomers, cobalt acetate in DMF : DMSO (1 : 1, v/v), and ionic liquid [BMIM] [BF_4_] were mixed in a vial. The porogen ratios of [BMIM] [BF_4_] : DMF : DMSO were 1 : 1:1 (v/v) and 10 : 1:5 (v/v) for preparation of MMIPs with TRIM and EDMA as crosslinkers, respectively. The prepolymerization mixture was homogenized using the sonicator for 5 min and added with 1% of AIBN as the radical initiator and further homogenized for 10 min. The homogenized mixture was then injected into the activated silicosteel® tubing (Section 2.3) using the adapter syringe. Both the column ends were closed using close-end nuts (Supelco) for in situ copolymerizations in the oven at 60 °C for 18 h.

After the polymerization process, monolithic columns were connected with the HPLC pump and washed with methanol: acetic acid (9 : 1, v/v) mixture for 4 h with a flow rate of 0.05 mL/min to remove residual monomers, porogen, (R)-(+)-citronellal template, and Co (II) used in the MMIP monolith preparation. To confirm the removal of (R)-(+)-citronellal template, the spectra of washing solution effluent collected every 1 h were recorded at the wavelength of 253 nm. A similar procedure was also applied to estimate the amount of Co (II) removed from the MMIP column. The calibration curve of Co (II) was constructed by dissolving Co (II) acetate in the mixture of methanol: acetic acid (9 : 1, v/v), which was detected at 517 nm by the UV-Vis spectrophotometer.

The morphological observation of the produced MMIP monolithic columns was carried out using scanning electron microscopy (SEM). Subsequently, a permeability test was performed by measuring the column's backpressure by employing the HPLC pump with the solvent of acetonitrile: water (50 : 50, v/v) as the mobile phase at a constant flow rate of 0.05 mL/min. The detected pressure drop corresponding to the monolith's permeability was further calculated according to the Darcy equation [24, 27].

Metal-mediated nonimprinted polymer (MNIP), molecularly imprinted polymer (MIP), and nonimprinted polymer (NIP) were prepared with the same method. The difference was the absence of template in preparing MNIP, metal pivot in MIP, both template and metal pivot in NIP.

### 2.5. Separation of Rac-Citronellal Using HPLC with MMIP as the Stationary Phase

MMIP monolithic column was paired with HPLC system for the separation of *rac*-citronellal samples. The mixture of ACN with water was used as the mobile phase at appropriate compositions depending on the elution techniques (isocratic and gradient elutions). The process was carried out at room temperature, at a flow rate of 0.04 mL/min, and an injection volume of 2 *μ*L and detected with the UV detector at 253 nm. Chromatogram profiles for each sample were then observed and calculated for the resolution of each peak. The same procedure was carried out for MNIP, MIP, and NIP as the stationary phase.

## 3. Results and Discussion

### 3.1. Synthesis and Characterization of Metal-Mediated Molecularly Imprinted Polymer (MMIP) Monolith

Silicosteel column was pretreated in advance through silanization before it was used as the column housing for MMIP, MNIP, MIP, and NIP monoliths. The silanization process was carried out by hydrolyzing the column with acid and base solutions (NaOH and HCl). MAPS was added to the column to facilitate covalent bonds forming between the inner wall surfaces of the silicosteel column and polymer. Silanization reaction on the inner wall surface of the silicosteel column occurred between the silanol group and the methoxy group of MAPS.

From the synthesis, two kinds of monoliths with the crosslinkers of TRIM and EDMA were obtained. The polymerization of MMIP, MNIP, MIP, and NIP monoliths was conducted in situ or using the one-pot method inside microbore columns made of the silicosteel with an inner diameter of 1 mm. The method was preferred due to the following advantages: it showed a higher enantiomer resolution to separate chiral compounds, faster synthesis than other polymerization methods, provided reasonable control to achieve desired characteristics of the monolith through optimization of chemical and physical parameters, and was easy to perform [28]. A microbore monolith column (1 mm i.d) was used for its easiness to be paired with the HPLC system without modification as compared to the capillary column, and small quantities of enantiomer samples were also another advantage. Additionally, the use of fewer solvents made it more economical and environmentally friendly. The synthesis of monolith inside large-diameter columns is still challenging due to the unequal heating along the column and gravitational settling effects [29].

**Figure 2:**
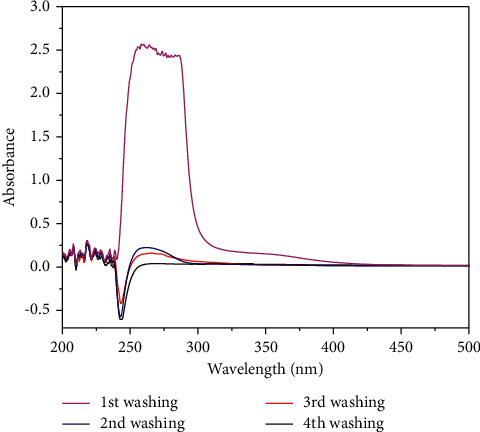
UV spectra of (R)-(+)-citronellal after removed from the MMIP A column using the HPLC pump with the eluent mixture of methanol and acetic acid (9 : 1, v/v) at a flow rate of 0.05 mL min^−1^. The measurement wavelength was 253 nm, and every step of washing was 1 h.

The template of (R)-(+)-citronellal and Co(II) used during the MMIP A monolith synthesis had been removed prior to its application as a chiral stationary phase (CSP) in HPLC by eluting/washing the MMIP monolithic column with a mixture of methanol and acetic acid (9 : 1, v/v). As shown in Figure 2, the template of (R)-(+)-citronellal was entirely removed from the MMIP A column after the 4^th^ washing as indicated by zero absorbance in the spectra.

Several studies have reported that the metal pivot is simultaneously released with the template [12, 30, 31]. According to Table 1, the Co(II) ion amount in the MMIP A monolith preparation was 8560 ppm, and the total Co (II) removed from the polymer was about 94.2% (Table 2) after washing the column with the mixture of methanol and acetic acid (9 : 1) for 4 h. The remaining 5.8% of cobalt is perhaps still present in the MMIP A monolith, and some may be lost because it is not bound to the monolith. The quantification of (R)-(+)-citronellal template and Co (II) on the MMIP B column was conducted in a similar way as in the MMIP A. It was found that a longer washing time of 8 h was required for the complete removal of the template and the metal pivot. This difference may be due to the higher amount of both components during the preparation of the MMIP B monolith, as shown in Table 1. As shown in Figure 1, after template removal, the imprint of the template remained in the polymer matrix. According to the imprinted matrix template, a pair of enantiomers passing through the polymer monolith would show the elution sequence. The enantiomer used as the template would be retained longer in the monolith column [32, 33].

The initial amount of Co (II) used for the MMIP monolith A synthesis was 8560 ppm, every step washing was 1 h, detection wavelength was 517 nm, and the washing solution was a mixture of methanol and acetic acid (9 : 1, v/v).

**Figure 3:**
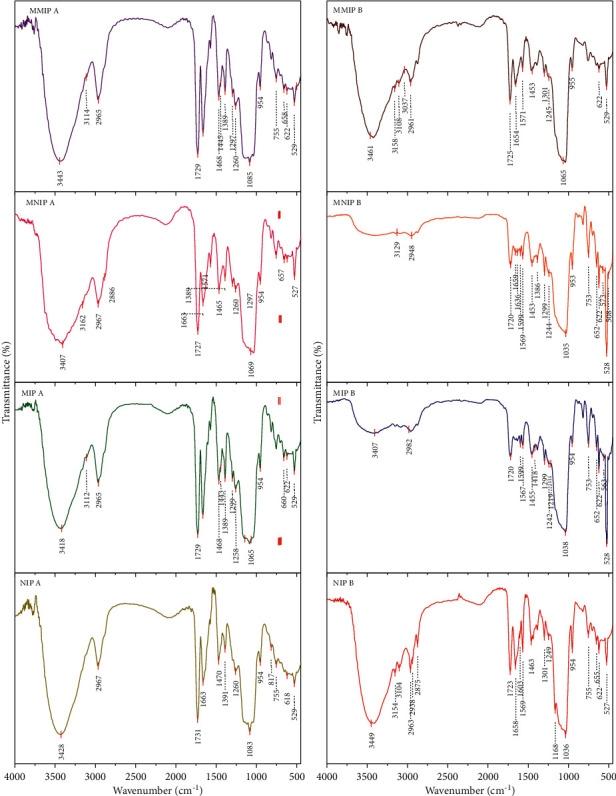
FTIR spectra of MMIP, MNIP, MIP, and NIP monoliths. The A and B notations correspond to the monoliths with the crosslinker of TRIM and EDMA, respectively (see Table 1).

The synthesized monoliths were subsequently characterized by FTIR. It was found that the IR spectra (Figure 3) of the monoliths prepared using TRIM and EDMA crosslinkers did not show different characteristics. However, there was a difference in the absorption sharpness at 1729 cm^−1^. MMIP A monolith had a sharper absorption with a higher % transmittance than MMIP B monolith absorption. This was because TRIM with 3 methacrylate groups had 3 carbonyl groups, while EDMA with 2 methacrylate groups had 2 carbonyl groups vibrating in that area. The more carbonyl groups in the monolith, the stronger the absorption shown by the FTIR spectra. All monoliths exhibited identical characteristic absorption at 3407–3445 cm^−1^ (OH group), except for MNIP B and MIP B, which showed weak absorption intensities. Other characteristic adsorptions appeared at 1725–1731 cm^−1^ (C=O group), 1642–1663 cm^−1^ (C=C group), and 1109–1159 cm^−1^ (C–O group). The result also showed absorptions around 527 cm^−1^ and 756 cm^−1^ associated with the deformation of the C–N–C group and C–H aromatic substitution from the 4-VP functional monomer. The metal presence in the monolith could be observed around the 2050–1755 cm^−1^ (Co-H adsorption), 610 cm^−1^ (linear Co-N), and 565 cm^−1^ (Co-O bending) regions and associated with the coordination of Co (II) with the atomic donor of the ligand [34, 35].

**Figure 4:**
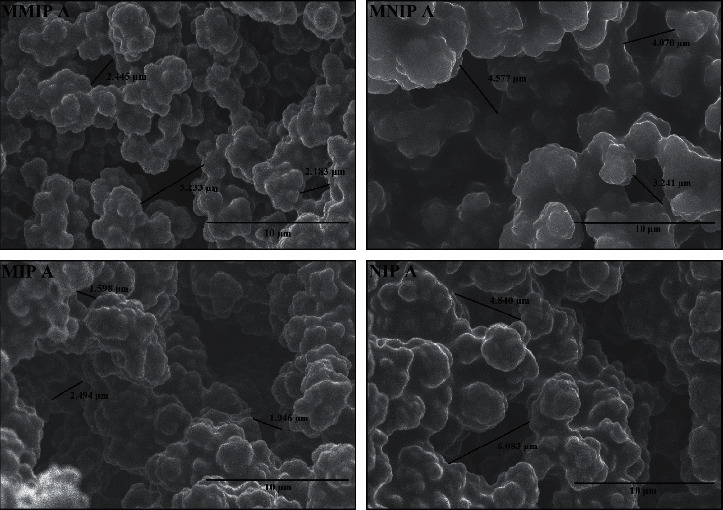
SEM images of monolith prepared with TRIM as the crosslinker at 5000x magnification. The monolith composition can be seen in Table 1.

**Figure 5:**
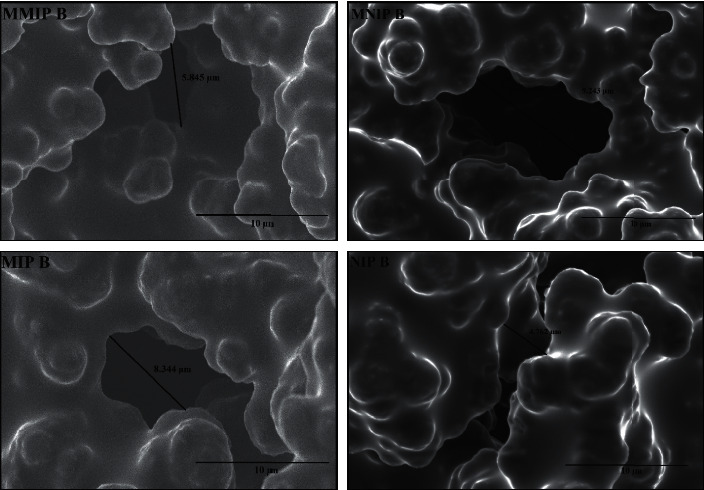
SEM images of monolith prepared with EDMA as the crosslinker at 5000x magnification. The monolith composition can be seen in Table 1.

The surface morphology of the monolith polymer was observed using SEM (Figures 4 and 5). The monolith morphological structures of MMIP, MNIP, MIP, and NIP showed dense globules formation with interconnected pores forming a continuous porous material. The spaces between globules allowed a flow to pass the through-pore, which could cause the convective flow of the mobile phase. The SEM images indicated no significant difference between MMIP or MNIP monolith contained Co(II) as the metal pivot compared to the NIP and MIP monolith without the metal pivot. However, the comparison on the surface morphology of monoliths prepared with different crosslinkers, TRIM (Figure 4) and EDMA (Figure 5), showed significant morphological differences. The use of EDMA resulted in a monolith with larger globules and through-pore sizes than that of TRIM. The result indicated that the composition of functional and crosslinking monomer, porogen, and metal as pivot significantly affected the morphology of the synthesized monoliths [15, 36].

Permeability measurement was carried out to confirm the mobile phase flow passes through the monolith's pores channel. As shown in Table 3, monoliths imprinted without Co (II) ions (MIP A and MIP B monoliths) showed a high permeability, with the MIP A monolithic column exhibiting the highest permeability value compared to the other synthesized monoliths. The increase in the permeability of the monolithic column was proportional to the larger through-pore size. In other words, the use of metal ions as a pivot in the prepolymerization mixture generated smaller through-pore sizes of the resulting monoliths.

Based on the SEM and permeability analysis, the MMIP B monolithic column demonstrated better quality than MMIP A. The globule and the flow-through pore size of MMIP B were larger than those of MMIP A, following the result of permeability analysis. The difference in the composition of template, AIBN, and metal affected the resulting monolith characteristics. The exact amount of added AIBN initiator in the synthesis of MMIP A was higher than that in MMIP B, although it was proportional to the total amount of monomer and the template (equal to 1% w/v). It indicated that the more monomers for synthesis were, the more AIBN for the monomer solution mixture would be. The use of excessive initiators led to more polymer nuclei formed at the start of the polymerization process, each of which got bigger and was interconnected to form a network. As a result, the monolith was too dense and less elastic, causing MMIP A to have a smaller through-pore size than MMIP B.

Generally, the quantification of monoliths' pores size for HPLC application was performed by the inverse size exclusion chromatography (ISEC) as shown in our previous work [1, 24, 27, 29, 37]. Although this method is ideal for “wet” chromatography, column destruction cannot be avoided. Therefore, we did not apply this method in the present work. To estimate the pore size of the monolith (although it is not quantitative), we use SEM morphology and permeability only. If the column permeability is high, it means that the pore formation of the monolith is good because the solvent can pass the column faster via the flow-through pore of the synthesized monolith. The results of the permeability analysis given in Table 3 confirmed that MMIP column A has lower permeability than MMIP column B. It means that the ability of MMIP column A to pass solvents is lower than that of MMIP column B. The MMIP A column produces a back pressure of 0.5 MPa, while the MMIP B column has a lower back pressure of 0.2 MPa. From the surface morphology analysis using the SEM data (Figures 4 and 5), the MMIP A column has a flow-through pore in the range of 2.18–5.24 *μ*m, while the MMIP B column is in the range of 5.85–9.24 *μ*m.

### 3.2. Application of MMIP Monolith as the Chiral Stationary Phase

The monolithic columns have a more dominant mesoporous size than micropores or macropores [1, 24]. If the macropores are too prevalent, the sample and mobile phase will come out from the column faster; thus, the separation is usually less optimal or causes unsatisfactory completion of sample separation. Such monoliths are less favorable for the enantio-separation of small molecules such as *rac*-citronellal essential oil. Mesopores provided sufficient space for interaction and adequate binding capacity between samples with the stationary phase [27], which was beneficial for separation research in biochemistry, environmental sciences, and pharmacy [37]. TRIM and EDMA have been widely used as a crosslinker monomer in the manufacture of imprinted monoliths [10, 38]. Interaction of template with functional monomer is considerably more prevalent in TRIM than in EDMA as a crosslinker. In other words, the interaction of the EDMA-template is more dominant than the TRIM template. This condition would affect the selectivity of imprinted monoliths [39].

**Figure 6:**
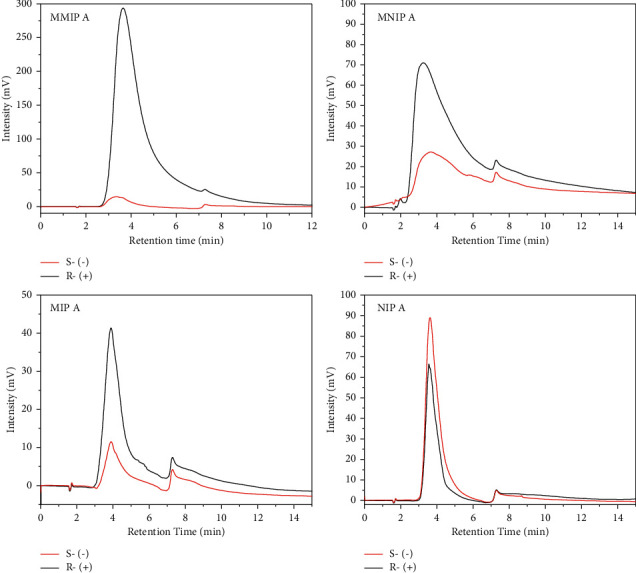
Comparison of MMIP, MNIP, MIP, and NIP monoliths with TRIM as a crosslinker for the elution of (R)-(+)-citronellal and (S)-(−)-citronellal. The flow rate of 0.04 mL/min and the mobile phase of 10% acetonitrile in water under the isocratic method were applied.

According to data described in Table 4 and Figure 6, monoliths prepared with templates and metal pivot (MMIP A, TRIM as a crosslinker) showed that (R)-(+)-citronellal was retained in the stationary phase longer than (S)-(−)-citronellal. On the other hand, (S)-(−)-citronellal was retained longer in MNIP A and NIP A column (monoliths without templates), while MIP eluted (R)-(+)-citronellal and (S)-(−)-citronellal with no significant difference in retention time. Therefore, it can be concluded that adding metal and template in the monolith is crucial and influential to separate enantiomer compounds.

**Figure 7:**
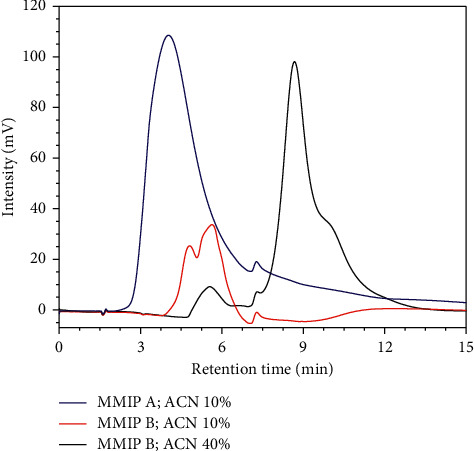
The effect of different crosslinkers employed in the separation of *rac*-citronellal. The isocratic elution using ACN/water as the mobile phase with a flow rate of 0.04 mL/min was used.

Table 5 and Figure 7 show that the MMIP B column prepared with EDMA crosslinker displayed the ability to separate rac-citronellal compounds with a selectivity of 1.26 and a resolution of 0.50 in ACN/water (10/90, v/v) and 1.12 and 0.32 in ACN/water (40/60, v/v). On the contrary, the separation of *rac*-citronellal compounds with the MMIP A column was considered ineffective. Accordingly, we examined the imprinted monoliths with EDMA crosslinker for further experiments.

**Figure 8:**
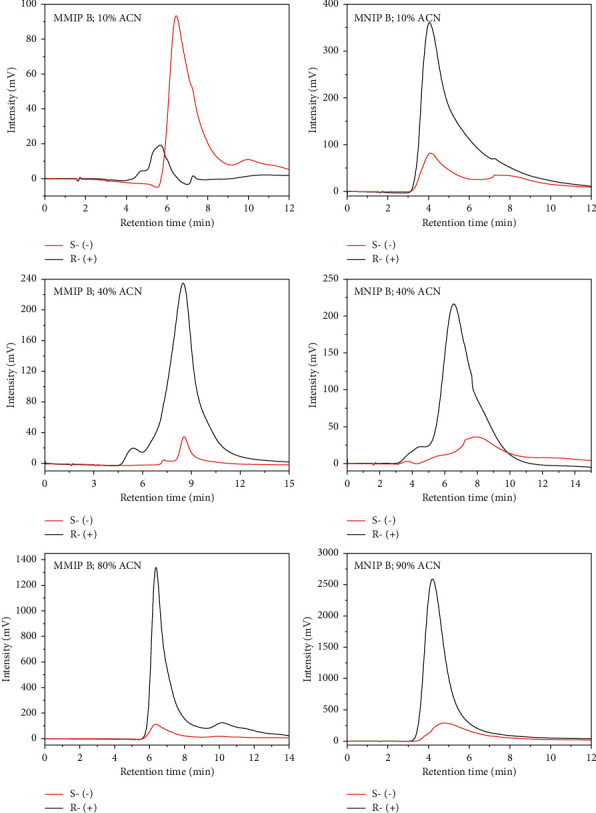
The effect of ACN concentration in water as the mobile phase to the retention time of (R)-(+)-citronellal and (S)-(−)-citronellal with MMIP B and MNIP B columns with the flow rate of 0.04 mL/min.

The variation in acetonitrile composition as the mobile phase was also investigated by MMIP B and MNIP B column, affecting the production capacity factor (**k**').  Figure 8 shows the CSP monolith column with various acetonitrile compositions indicating a good separation. Some (S)-(−)-citronellals retained longer than (R)-(+)-citronellal in the column as the compound was more distributed to the stationary phase than to the mobile phase. MNIP B column did not use templates during the synthesis process; thus, the enantiomeric pairs (R)-(+)-citronellal and (S)-(−)-citronellal exhibited the same ability to interact with polymer matrix when they were eluted through the column. This finding revealed that the use of acetonitrile at a lower ratio than water in the mixture of mobile phase caused the citronellal enantiomers to have a significant difference in residence time. The supporting data in Table 6 confirmed this finding. The use of the MMIP B column in different concentrations of ACN of 10% and 40% resulted in different retention times at 0.78 and 0.60 minutes, respectively.

Monolith columns imprinted by templates provide a more substantial recognition site as the stationary phase than those without templates, leading to better selectivity [10]. Thus, the *rac*-citronellal separation was optimized with the elution gradient method on MMIP B which was considered as the best column than the other 7 monolithic columns. CSP column had a 1 : 1 template to Co (II) ion ratio and a 1 : 6 template to functional monomer ratio.

**Figure 9:**
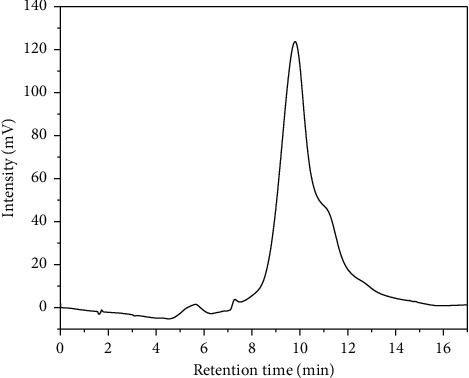
Chromatogram of *rac*-citronellal separation using MMIP B column. Mobile phase (A) H_2_O and mobile phase (B) acetonitrile 90% in (A), gradient elution: 70%–75% B for 8 mins, 75%–85% for 9 mins; detection wavelength at 253 nm; flow rate 0.04 mL/min.

**Figure 10:**
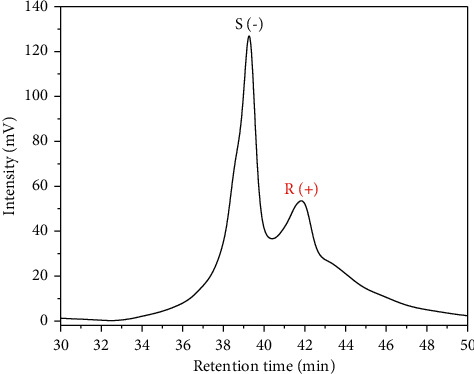
Chromatogram of *rac*-citronellal separation using MMIP B column. Mobile phase (A) H_2_O and mobile phase (B) acetonitrile 90% in (A), gradient elution: 30–60% B for 12 mins, 60–70% B for 18 mins, 70–20% for 30 mins, detection wavelength at 253 nm; flow rate 0.04 mL/min.

The isocratic method was not efficient in separating the enantiomeric *rac*-citronellal compounds; therefore, the elution gradient method was performed at the same flow rate (0.04 mL/min). A lower concentration of acetonitrile used as the mobile phase made the separation more effective. Consequently, the elution gradient method required water as the dominant mobile phase. The first peak started to appear at 6 minutes and the second at 10 minutes. However, the resolution (*Rs*) was not good as indicated; only a small split peak appeared (Figure 9 and Table 7). Based on the obtained chromatogram in Figure 9, the elution gradient method had to be modified to produce better resolution (Rs) of *rac*-citronellal, as shown in Figure 10 and Table 7.

The MMIP B as CSP column (Figure 10) exhibited a different performance in the *rac*-citronellal separation under the gradient elution method. The compound was eluted longer in the column and came out at a retention time of 38 mins, confirming that the synthesized monolith column interacted strongly with water as the solvent. A dominant water solvent used as HPLC mobile phase led to longer elution of *rac*-citronellal in the column. The monolith column contained several O donor atoms from a TRIM/EDMA crosslinker monomer and N donor atoms from the 4-VP functional monomer. As a result, protic solvents such as water enabled interaction forces among the hydrogen bonding molecules on the column and the water solvent. This finding is in accordance with Bodoki et al. [12], explaining the selection of functional monomer, crosslinker monomer, and porogen type in the monolith preparation. The consistency of *rac*-citronellal separation using monolith columns as chiral stationary phases (CSPs) in high-performance liquid chromatography (HPLC) is challenging to maintain. Therefore, it was complicated to obtain separation with an excellent resolution of *rac*-citronellal samples. The challenge of using MMIPs as CSPs in HPLC was a low separation efficiency due to slow interaction kinetics and heavy peak broadening [40].

## 4. Conclusions

Chiral stationary phase column of poly-MMIP-(4VP-EDMA-IL) monolith is more effective than poly-MMIP-(4VP-TRIM-IL) monolith for separating enantiomeric compounds of the *rac*-citronellal essential oil. The use of Co(II) as the metal pivot in the monolith column produces a denser material structure, larger and stiffer globules, and larger size of flow-through pore than that without Co(II) metal mediation.

## Figures and Tables

**Table 1:** Composition for the preparation of MMIP, MNIP, MIP, and NIP monolithic columns.

Columns	Molar ratio (T : M : Co : C)	T (mmol)	M/4-VP (mmol)	Co(II) (mmol)	C (mmol)	[BMIM] BF_4_ (mL)	DMF (mL)	DMSO (mL)
					**TRIM**	**1**	**1**	**1**
MMIP A	1 : 5:1 : 20	0.067	0.33	0.067	1.34	0.30	0.30	0.30
MNIP A	−:5 : 1:20	–	0.33	0.067	1.34	0.30	0.30	0.30
MIP A	1 : 5:-:20	0.067	0.33	–	1.34	0.30	0.30	0.30
NIP A	−:5:-:20	–	0.33	–	1.34	0.30	0.30	0.30
					**EDMA**	**10**	**1**	**5**
MMIP B	1 : 6:1 : 18	0.10	0.60	0.10	1.80	0.82	0.08	0.40
MNIP B	−:6 : 1:18	–	0.60	0.10	1.80	0.82	0.08	0.40
MIP B	1 : 6:-:18	0.10	0.60	–	1.80	0.82	0.08	0.40
NIP B	−:6:-:18	–	0.60	–	1.80	0.82	0.08	0.40

T: template; M: functional monomer; Co: cobalt acetate; C: crosslinker. Notation of A and B in columns' name corresponds to the monoliths with crosslinker of TRIM and EDMA, respectively. The bold values showed the porogen volume ratio of [BMIM] BF4, DMF, and DMSO.

**Table 2:** Analysis of the cobalt acetate content after washing sequence from the MMIP A column.

Sample	Co (II) absorbance	Concentration (ppm)
1^st^ washing solution	0.086	1770
2^nd^ washing solution	0.105	2086.67
3^rd^ washing solution	0.106	2103.33
4^th^ washing solution	0.106	2103.33
Total		8063.33

**Table 3:** Column permeability of MMIP, MNIP, MIP, and NIP monoliths.

Monolithic column	Permeability (m^2^)
MMIP A	1.74 × 10^−13^
MNIP A	2.90 × 10^−13^
MIP A	4.35 × 10^−13^
NIP A	2.18 × 10^−13^
MMIP B	2.49 × 10^−13^
MNIP B	1.45 × 10^−13^
MIP B	2.90 × 10^−13^
NIP B	2.18 × 10^−13^

**Table 4:** The isocratic elution of (R)-(+)-citronellal and (S)-(−)-citronellal using 10% ACN (in water) using MMIP, MNIP, MIP, and NIP monoliths with TRIM as a crosslinker.

Monolithic column	Sample	*t*_0_ (min)	*t*_r_ (min)	*k′*
MMIP A	(R)-(+)-citronellal	1.72	3.64	1.12
	(S)-(−)-citronellal	1.72	3.35	0.95
MNIP A	(R)-(+)-citronellal	1.72	3.25	0.89
	(S)-(−)-citronellal	1.72	3.65	1.12
MIP A	(R)-(+)-citronellal	1.72	3.89	1.26
	(S)-(−)-citronellal	1.72	3.89	1.26
NIP A	(R)-(+)-citronellal	1.72	3.56	1.07
	(S)-(−)-citronellal	1.72	3.62	1.10

**Table 5:** Enantiomer separation with a variation in mobile phase composition on monolithic columns prepared with a different crosslinker.

Column	ACN (%, v/v)	*t*_*r*_^1^ (min)	*t*_*r*_^2^ (min)	*t*_0_ (min)	**k**′_*t*_*R*_1_′	**k**′_*t*_*R*_2_′	*α*	*R*_*s*_
MMIP A	10	4.03	-	1.72	1.34	-	-	-
MMIP B	10	4.81	5.63	1.72	1.80	2.27	1.26	0.50
	40	8.67	9.51	1.72	4.04	4.53	1.12	0.32

**Table 6:** Capacity factor (k') on the MNIP B column using a mobile phase with different ACN compositions using the isocratic elution method.

Column	ACN in water (%, v/v)	Sample	*t*_0_ (min)	*t*_r_ (min)	*k'*
MMIP B	10	(R)-(+)-citronellal	1.72	5.67	2.30
		(S)-(−)-citronellal	1.72	6.45	2.75
	40	(R)-(+)-citronellal	1.72	8.49	3.94
		(S)-(−)-citronellal	1.72	8.55	3.97
	80	(R)-(+)-citronellal	1.72	6.42	2.74
		(S)-(−)-citronellal	1.72	6.37	2.70
MNIP B	10	(R)-(+)-citronellal	1.72	4.04	1.35
		(S)-(−)-citronellal	1.72	4.11	1.39
	40	(R)-(+)-citronellal	1.72	8.55	3.97
		(S)-(−)-citronellal	1.72	7.95	3.63
	90	(R)-(+)-citronellal	1.72	4.19	1.44
		(S)-(−)-citronellal	1.72	4.80	1.79

**Table 7:** Enantiomer separation of rac-citronellal on the MMIP B column using the gradient elution method.

Gradient program	*t*_*r*_^1^ (min)	*t*_*r*_^2^ (min)	*t*_0_ (min)	**k**_**t**_**R**_1_′	**k**_**t**_**R**_2_′	*α*	*R*_*s*_
X	9.80	10.79	1.72	4.70	5.28	1.12	0.66
Y	39.26	41.83	1.72	21.84	23.33	1.07	1.71

*t*_*r*_^1^ and *t*_*r*_^2^ correspond to the retention time of (S)-(−)-citronellal and (R)-(+)-citronellal, respectively; X: mobile phase and gradient program are the same as in Figure 9; Y: mobile phase and gradient program are the same as in Figure 10.

## Data Availability

The data used to support the findings of this study are available from the corresponding author upon request.
